# Supplementary material for: Agroecosystem resilience. A conceptual and methodological framework for evaluation
Source: PLoS One. 2020 Apr 22;15(4):e0220349. doi: 10.1371/journal.pone.0220349 (PMC7176124; doi:10.1371/journal.pone.0220349)
Supplement: Attachment — Submitted filename: Response to reviewer.docx [file pone.0220349.s003.docx]

Reviewer comment responses

Reviewer #2: Again, I applaud the inclusion of agrarian structure and peasant agency into a statistical analysis. I think that the model is well defined, interesting, and useful. However, the way that resilience is conceptualized and framed theoretical remains highly problematic. The authors write: "resilience has among other aims, the achievement of food sovereignty" (page 6), later (page 7) the authors write: "resilience allows communities to have greater control over management and governance of food & agricultural system..." and also on page 7 "resilience is key to adaptation". Then later, on page 7, the authors suggests that "from an agroecological perspective" resilience is basically the same definition of food sovereignty. This framing is problematic. I wonder if this is a language issue, but I believe the authors have confused the dependent and independent variables. Are the authors trying to measure resilience? If so, resilience is the dependent variable. They should not then be identifying what factors resilience leads to, and then measuring those factors as if they were the dependent variable, but go on to make a claim about resilience. I am more aware of research that suggests that greater peasant agency, greater control of resources, and a higher degree of food sovereignty leads to (as causal factors) higher resilience. If resilience is what we are trying to measure/explain, then we must frame resilience as the dependent, not the causal, factor. Thus, it is not correct to say that "resilience is key to adaptation", but that adaptability is the key factor that contributes to resilience. Similarly, greater control over food and agrarian systems is a factor that helps to develop (causally) resilience capacity.

The authors thank the reviewer for the observations offered. It is correct that the writing in one of the paragraphs on page 7 leads to an understanding of resilience as an independent variable or causal factor. Our objective in this article is to consistently identify resilience as the dependent variable. Rural communities with a greater degree of food sovereignty, greater control of resources and the capacity to manage these, as well as true participation in political processes that allow for the transformation of the conditions of inequality, can develop and/or strengthen their resilience. As a means of response, we have complemented both the introduction as well as page 7, to clarify that resilience is considered a dependent variable.

Also, as an aside, I would very much like to see an argument of the paper earlier on -- preferably in both the abstract and the introduction. I think it is something like: "Agrarian structure and peasant agency are important elements in determining resilience capacities. This paper finds that when agrarian structure is more equal, and when peasant agency is more strongly developed through political organizations, training, and involvement of women, then there is greater capacities for generating transformational resilience that improves the dignity of peasant livelihoods.

The authors accept this comment and incorporate the ideas into the summary as well as the introduction (page 2, paragraph 1; page 4, paragraph 2; page 7, paragraph 2).
